# Staged correction of an equinovarus deformity due to pyoderma gangrenosum using a Taylor spatial frame and tibiotalar calcaneal fusion with an intramedullary device

**DOI:** 10.1007/s11751-011-0119-y

**Published:** 2011-08-24

**Authors:** Jaime L. Bellamy, Courtney A. Holland, Mark Hsiao, Joseph R. Hsu, STReC (Skeletal Trauma Research Consortium)

**Affiliations:** 1 Brooke Army Medical Center, Fort Sam Houston, San Antonio, TX USA; 2 William Beaumont Army Medical Center, El Paso, TX USA; 3 Creighton University, Omaha, NE USA; 4 United States Army Institute of Surgical Research, Fort Sam Houston, San Antonio, TX USA

**Keywords:** Pyoderma gangrenosum, Equinovarus, Staged fusion, Taylor spatial frame

## Abstract

Pyoderma gangrenosum is a rare autoinflammatory syndrome manifested by skin lesions eventually creating ulcers. Surgical management can lead to scarring and contracture at the site of the lesion due to the pathergy phenomenon. A 43-year-old woman presented with a 5-year history of severe equinovarus deformity due to chronic pyoderma gangrenosum on her posteromedial ankle. She underwent a staged fusion. A gradual “closed” correction was performed in a Taylor spatial frame for 8 weeks in order to obviate the need for a surgical release in the area of the ulcer. She was ambulatory and full weight-bearing within 4 weeks of her frame removal. She maintained her correction with an accommodative foot orthosis until she had an uneventful tibiotalar calcaneal fusion with an intramedullary device. This case represents the success of using a Taylor spatial frame for staged fusion involving soft-tissue correction of severe, rigid equinovarus deformity due to pyoderma gangrenosum.

## Introduction

Pyoderma gangrenosum (PG) is a rare autoinflammatory syndrome of unknown etiology. In up to 70% of cases, PG is associated with underlying systemic disease, such as inflammatory bowel disease, rheumatoid arthritis or malignancy [1]. PG is a noninfectious neutrophilic dermatosis that causes recurrent painful inflammatory ulcerations [2]. Lesions are commonly found on the dorsal surface of the feet and legs but may occur on the arms, chest, stoma and the face. Skin lesions are tender, contain fluctuant nodules with surrounding erythema and spread peripherally creating an ulcer. Additionally, epidermal necrosis gives ulcers a blue hue [3]. Successful treatment most importantly involves addressing the underlying disease.

PG management includes medical and surgical modalities. The unknown etiology and pathophysiology have made the treatment for PG difficult [4]. Management of PG is divided into systemic and topical medical therapy and local wound care with surgical debridements [5]. Recently, infliximab and tacrolimus have been shown to be effective systemic and topical medical treatments for PG, respectively [6, 7]. Wound debridement has been beneficial but has the potential to worsen the lesion due to the pathergy phenomenon. Pathergy is the development of new skin lesions at sites of minor trauma, biopsy or needle sticks [8]. This phenomenon of PG limits and complicates surgical procedures as repeated debridements can lead to scarring and contracture at the site of the lesion.

Contractures over tendons of the foot can lead to foot deformities. The posterior tibialis and the flexor digitorum longus muscles pull the foot into an equinus and cavovarus position. Severe scarring after burns, crush injuries or venous stasis may pull the foot into the cavovarus position [9]. Equinovarus foot deformities are most often due to spasticity of the posterior tibialis muscle and/or the anterior tibialis muscle, which are active in the stance phase and swing phase of gait, respectively. For body weight to advance onto the forefoot, a 5° angle is necessary and is often absent with a plantar flexion contracture [10]. The most common surgical treatments for foot deformities due to soft-tissue malfunction include tendon releases, tendon transfers and arthrodesis [11, 12]. We describe a case using gradual “closed” correction using the Taylor spatial frame (TSF; Smith & Nephew, Memphis, Tennessee) technique.

## Case report

**Fig. 1:**
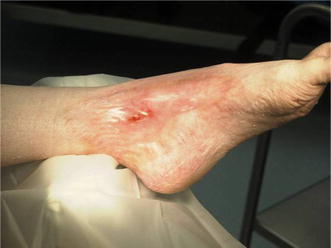
Pre-operative pyoderma gangrenosum

**Fig. 2:**
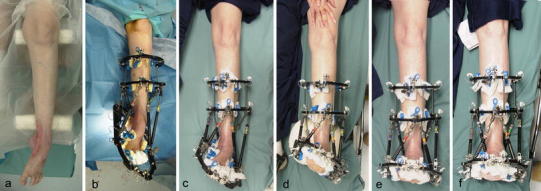
Pre-operative (**a**) and intra-operative placement of TSF (**b**). Twenty days (**c**), 5 weeks (**d**), 6 weeks (**e**) and 8 weeks (**f**) post-operative gradual correction in the TSF

A 43-year-old woman presented with a severe (80° equinus), rigid equinovarus foot deformity due to chronic PG. She had a 5-year history of a large, nonhealing ulcer centered over the left medial malleolus and underwent multiple debridements resulting in her worsening scar tissue (Fig. 1). The patient gradually developed a severe equinovarus deformity (Fig. 2a). It progressed to the point that she was non-weight-bearing for 3 years prior to this intervention. Pre-operative computed tomography demonstrated loss of articular space along the posterior one-third of the tibial plafond and talus.

The likelihood of an eventual fusion was discussed with the patient. She desired to stage the fusion. In her case, a one-stage arthrodesis would have likely necessitated an open posteromedial release directly through the zone of inflammation. Furthermore, a single-stage approach may have required a complete talectomy with resultant shortening to safely achieve the correction without neurovascular compromise.

To avoid an open surgical release to achieve correction, which could exacerbate her PG, a gradual “closed” correction was performed using a TSF. The posteromedial skin edge was designated as the “structure at risk” so it and the underlying tibial neurovascular bundle could be stretched no more than 1 mm a day.

### Surgical technique

A standard equinus correction frame was constructed with a TSF. The frame was fixed to the extremity with a combination of wires and hydroxyapatite (HA)-coated half-pins. Additional points of fixation were added at each level due to the patient’s extreme disuse osteopenia. Despite this severe osteopenia, HA-coated half-pins were utilized in a crossing manner in the calcaneus with adequate purchase [13]. We elected not to fix the talus with a transosseus wire, since this would have placed the wire directly through the zone of inflammation. We performed no open or percutaneous soft-tissue releases.

We utilized the TSF software to create a “virtual hinge” at the center of rotation of the ankle. This “virtual hinge” allowed precise correction of the deformity without the need for frame adjustments beyond routine strut changes as described by the software.

The TSF software allows for the identification of a “structure at risk” (SAR). The SAR can then be used to determine the pace of the correction. We defined the posteromedial skin edge as the SAR, so we could stretch this slowly at 1 mm/day. This slow, gradual correction took 8 weeks (Fig. 2b–f). We held the foot in an overcorrected position for an additional 4 weeks. At frame removal, the patient was placed in a custom accommodative foot orthosis for maintenance of the correction.

**Fig. 3:**
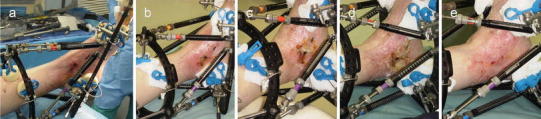
Intra-operative PG (**a**). Twenty days (**b**), 5 weeks (**c**), 6 weeks (**d**) and 8 weeks (**e**) post-operative in the TSF showed no exacerbation of PG

**Fig. 4:**
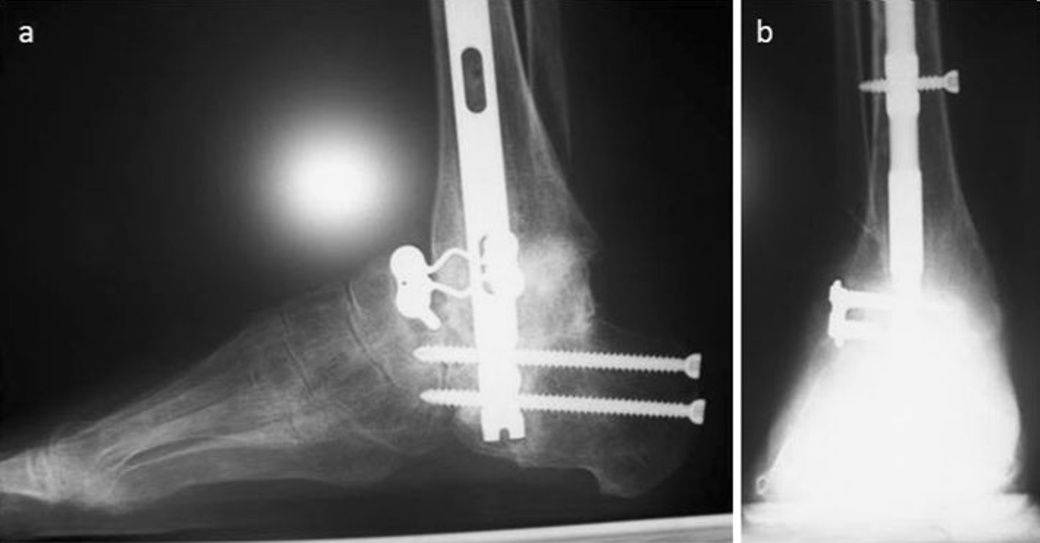
Lateral (**a**) and AP (**b**) post-operative tibiotalar calcaneal fusion with an intramedullary device

The patient’s longstanding deformity was corrected without additional incisions beyond those necessary for percutaneous placement of wires and half-pins. No open soft-tissue releases were required. There were no deep pin tract infections. The half-pins in the calcaneus did not loosen and had adequate extraction torque at the time of removal. There was no neurovascular compromise. The patient did not have an exacerbation of her PG (Fig. 3a–e). The patient was ambulatory and full weight-bearing within 4 weeks of her frame removal. She maintained her correction with an accommodative foot orthosis until she had an uneventful tibiotalar calcaneal fusion with an intramedullary device (Fig. 4a–b). She is currently more than 18 months from her “closed” procedure with the TSF and 6 months from her ankle arthrodesis. She has healed her fusion without signs of deep infection. She has a minimal leg length discrepancy (<1 cm) and has no pain in the ankle or hind foot.

## Discussion

PG is a rare autoinflammatory syndrome of unknown etiology in which treatment is complicated by the pathergy phenomenon: debridements that scar can lead to contractures and subsequent foot deformities. Tendon releases, tendon transfers, arthrodesis and ring external fixators, like the TSF, may be used to correct these deformities.

The TSF is safe and minimally invasive. The slow, gradual correction obviated the need for an open surgical procedure to obtain correction. Due to the pathergy phenomenon, surgical release of tendons would likely exacerbate soft-tissue injury in patients with PG. The TSF is an external fixation device that achieves correction by stretching soft tissues gradually [13–15], including foot and ankle deformities [14, 16, 17], over time. There are no reported cases in the literature regarding surgical correction of complex rigid equinovarus foot deformities due to PG.

The benefits of a gradual correction over acute correction with fusion in our case were twofold. First, this eliminated the need for a medial incision through the ulcer to release the scar contracture. Second, this allowed for correction to a plantigrade foot without neurovascular compromise. A safe acute correction would have necessitated unacceptable shortening and possible talectomy. In this case, the patient was ambulatory and full weight-bearing within 4 weeks after frame removal. This case highlights the TSF technique as a powerful tool for safe treatment for severe, rigid equinovarus deformity due to PG.
